# Consumer Perspectives on the Use of Artificial Intelligence Technology and Automation in Crisis Support Services: Mixed Methods Study

**DOI:** 10.2196/34514

**Published:** 2022-08-05

**Authors:** Jennifer S Ma, Megan O’Riordan, Kelly Mazzer, Philip J Batterham, Sally Bradford, Kairi Kõlves, Nickolai Titov, Britt Klein, Debra J Rickwood

**Affiliations:** 1 Discipline of Psychology Faculty of Health University of Canberra ACT Australia; 2 Centre for Mental Health Research National Centre for Epidemiology and Population Health The Australian National University Canberra Australia; 3 Rehabilitation, Aged and Community Services Psychology & Counselling Team University of Canberra Hospital Canberra Australia; 4 Department of Veteran Affairs Australian Government Canberra Australia; 5 Australian Institute for Suicide Research and Prevention School of Applied Psychology Griffith University Brisbane Australia; 6 MindSpot and School of Psychology Macquarie University Sydney Australia; 7 Health Innovation and Transformation Centre Federation University Australia Churchill Australia

**Keywords:** consumer, community, help-seeker, perspective, technology, artificial intelligence, crisis, support, acceptability

## Abstract

**Background:**

Emerging technologies, such as artificial intelligence (AI), have the potential to enhance service responsiveness and quality, improve reach to underserved groups, and help address the lack of workforce capacity in health and mental health care. However, little research has been conducted on the acceptability of AI, particularly in mental health and crisis support, and how this may inform the development of responsible and responsive innovation in the area.

**Objective:**

This study aims to explore the level of support for the use of technology and automation, such as AI, in Lifeline’s crisis support services in Australia; the likelihood of service use if technology and automation were implemented; the impact of demographic characteristics on the level of support and likelihood of service use; and reasons for not using Lifeline’s crisis support services if technology and automation were implemented in the future.

**Methods:**

A mixed methods study involving a computer-assisted telephone interview and a web-based survey was undertaken from 2019 to 2020 to explore expectations and anticipated outcomes of Lifeline’s crisis support services in a nationally representative community sample (n=1300) and a Lifeline help-seeker sample (n=553). Participants were aged between 18 and 93 years. Quantitative descriptive analysis, binary logistic regression models, and qualitative thematic analysis were conducted to address the research objectives.

**Results:**

One-third of the community and help-seeker participants did not support the collection of information about service users through technology and automation (ie, via AI), and approximately half of the participants reported that they would be less likely to use the service if automation was introduced. Significant demographic differences were observed between the community and help-seeker samples. Of the demographics, only older age predicted being less likely to endorse technology and automation to tailor Lifeline’s crisis support service and use such services (odds ratio 1.48-1.66, 99% CI 1.03-2.38; *P*<.001 to *P*=.005). The most common reason for reluctance, reported by both samples, was that respondents wanted to speak to a real person, assuming that human counselors would be replaced by automated robots or machine services.

**Conclusions:**

Although Lifeline plans to always have a real person providing crisis support, help-seekers automatically fear this will not be the case if new technology and automation such as AI are introduced. Consequently, incorporating innovative use of technology to improve help-seeker outcomes in such services will require careful messaging and assurance that the human connection will continue.

## Introduction

### Background

In 2016, the founder and executive chairman of the World Economic Forum, Klaus Schwab, wrote that “we stand on the brink of a technological revolution that will fundamentally alter the way we live, work, and relate to one another” [1]. Schwab was referring to the advent of the Fourth Industrial Revolution, which will exponentially build upon the simple digitalization seen in the Third Industrial Revolution through innovations that combine the physical, digital, and biological spheres.

One such innovation has been the development of artificial intelligence (AI). AI has been described as the ability of a computer or machine to mimic the capabilities of the human mind, such as learning from examples and experiences, recognizing objects, understanding and responding to language, making decisions, and solving problems [2]. Although AI is widely used in many applications, the awareness of AI’s use and functions is relatively low [3,4]. For example, a survey of 6000 adults across North America, Europe, the Middle East, Africa, and the Asia-Pacific revealed that 84% had recently used at least one AI-powered service or device (eg, email spam filters, predictive search terms, and personal assistants), but only 34% had identified that they had interacted with some sort of AI technology in the recent past [3].

### AI in Health and Mental Health

Importantly, in the fields of health and mental health, AI has been argued to have the potential to enhance existing services by facilitating diagnostics and decision-making, expand the reach and personalization of services to underserved populations and high-risk groups, and ease the human resources crisis in mental health care and support [5-8]. For example, machine learning (ML), a subset of AI that uses advanced statistical and probabilistic techniques to construct systems with the ability to automatically learn from large and varied data sources, is currently being explored to improve the detection and diagnosis of mental health and neurodegenerative conditions such as depression, suicidality, schizophrenia, and Alzheimer disease [9,10].

AI has even been viewed by some academics as representing the future of mental health research methodology because of its superior ability to recognize the complexity of disorders, heterogeneity of clients, and varied mental health contexts compared with traditional statistical approaches that tend to rely on *forecasting* with only a few variables [11,12]. AI can deal with and learn from large and complex data, including the concurrent analysis of multiple factors rather than traditional additive, interactive, and linear statistical models.

Although the current use of ML techniques for diagnosis in real-world mental health settings is limited because of the lack of clinical validation and readiness of ML applications [6,9,13], AI is already being used to support practitioners and clients in monitoring treatment progress and medication adherence, delivering remote therapeutic sessions, and providing intelligent self-assessments [6]. ML algorithms are also used on social media platforms and virtual assistants, such as Facebook, Google, and Apple, to flag suicidal content posted or voiced by users and direct them to relevant crisis support and emergency services based on the assessments of risk, sometimes with the help of counselors from collaborating crisis support services such as the Crisis Text Line in the United States, Canada, South Africa, Ireland, and the Trevor Project in Australia [14,15]. With increased reliance on mental health and crisis support services observed worldwide in response to the COVID-19 pandemic [16,17], it is clear that crisis support for personal crises, such as suicidality, mental health issues, and situational crises, is an essential part of the mental health and public health systems, where the use of new technologies could substantially enhance the much-needed capacity.

### Lifeline Context

In Australia, the national 24-hour crisis support service for the general community, Lifeline, featured heavily in the Australian Department of Health’s $10.4 (US $7.2) million national mental health communications campaign to encourage Australians to reach out for mental health support during COVID-19 [18]. This charitable service has been operating since 1963 and is currently delivered via telephone, SMS text messaging, and web-based chat modalities in 41 centers staffed by 3364 volunteers and paid crisis counselors across Australia [19,20]. In the 2019-2020 financial year, Lifeline serviced 989,192 calls (84.5% call answer rate), 39,680 SMS text messaging contacts, and 53,527 web-based chat conversations, leading to the creation of 43,431 self-harm and suicide prevention plans [19]. Notably, in the context of COVID-19, a 25% increase in service demand (increasing to 90,000 calls per month) was reported compared with that during the same time in the previous year [21]. Half of the calls received in this period were from people reporting difficulties associated with COVID-19, and in 2021, 1 in 5 calls went unanswered [22]. Internationally, COVID-19–related increases in helpline use have resulted in increased call wait time [23], which negatively affects service users’ experience. High call volumes have been cited as a major cause of staff burnout and attrition in this sector [24]. Within Lifeline, telephone crisis supporters’ psychological well-being has been found to significantly impact counseling ability and service delivery [25]. These statistics highlight that crisis support services, such as Lifeline, need to be familiar with the current and future uses of AI and how it can complement existing practices and enhance capacity, while not replacing vital human aspects of the therapeutic relationship, such as personal connection and trust [5,6,26].

### Consumer Acceptance of New Technologies

Despite the rapid advancement of technological innovations in health care, research on consumers’ acceptance of new technologies has been scarce. To the best of our knowledge, there has been no research on consumer perspectives of AI as applied to the fields of mental health and crisis support, representing significant knowledge and practice gaps in this area. A recent systematic review of 117 articles published from 2005 to 2016 on data mining for AI in health care analytics revealed that one-third of the reviewed research did not use expert opinions in any form [27], indicating that a significant proportion of researchers and key stakeholders (ie, patients, service users, carers, and families) may not be consulted in discussions about AI and its application in health care.

In addition, the few studies conducted specifically on consumer perspectives of AI have focused solely on its use in medical health contexts. Nascent research has shown that trust and understanding of AI are important factors in the acceptance of AI in medical applications [28,29]. For example, in a study of 307 adults in the United States, consumer concerns about technology, ethics (perceived privacy concerns, mistrust in AI mechanisms, and social bias), and regulatory processes (ie, unregulated standards and perceived liability issues) were found to contribute to the perceived risks of AI medical devices [28]. Consumers have also been found to be less likely to use medical health care if delivered via an automated computer that uses AI compared with a human provider, even in situations where the performance of AI was explicitly specified to participants as being superior to that of human providers [29]. The researchers attributed this to the psychological driver of *uniqueness neglect*, which they stipulated to occur when consumers believe that AI medical health providers are unable to take into account the uniqueness of their case to the same extent as human providers, suggesting this as a potential target point in consumer education about AI [29].

These concerns have been largely corroborated by reports from surveys of nationally representative and consumer samples. For instance, in a 2020 survey of 2575 Australians, perceptions of the adequacy of current regulations and laws to make AI use safe, the uncertain impact of AI on society and jobs, and reported familiarity and understanding of AI were found to strongly influence AI acceptance more broadly [30]. Interestingly, reports have also shown that consumers have low levels of trust, high levels of fears and concerns, and low levels of awareness or understanding of AI [4,30-35]. In particular, a strong preference for human-centered care and personal contact has been emphasized by participants [31,34,35].

### This Study

To date, research has focused on medical care applications, and the extent to which findings can be translated into AI applications in mental health and crisis support contexts remains unclear. With global investment in AI technology rising from 1.7 billion in 2010 to 14.9 billion in 2014 [36], research into consumers’ levels of awareness and support for AI-integrated mental health and crisis support, as well as their concerns and expectations around such support services, is needed to ensure responsible and responsive innovation. This is particularly pertinent for promoting effective communication around the risks and benefits of AI-integrated mental health and crisis support as well as the uptake of initiatives aimed at enhancing capacity and supporting the delivery of existing practices via new technologies such as AI.

A possible avenue for AI-integrated technology to promote increased service capacity and quality is to support the crisis counselor workforce (often volunteer-based) to feel better equipped to support help-seekers, train and support each other, and prevent staff burnout and attrition at an organizational level. Research shows that crisis counselors spend a considerable amount of time taking manual notes and cross-referencing these notes while actively trying to support help-seekers, which adds to their cognitive load [37]. AI-integrated applications could include the development of ML algorithms to automatically detect crisis callers’ levels of risk and distress based on validated voice or text features analyzed using speech recognition or natural language processing during contact. Help-seekers’ trajectories on highly relevant service-related outcomes (eg, connectedness and suicidality) could then be visually mapped in real time to support crisis support processes and practices. Crisis counselors (and their supervisors) could use this visual reference tool to more quickly identify key presenting crises, check whether the support provided has an appreciable effect on help-seeker outcomes, and tailor support accordingly. Such a tool would be of value to a service such as Lifeline because it receives requests for support from a very broad range of help-seekers and is expected to provide the same quality of care across these diverse groups and types of crises [38]. Recent research has found that not all help-seeker groups experience the same level of positive outcomes from, and satisfaction with, the Lifeline service [39]. AI-integrated technological support may be able to provide supplementary information not captured by current service measures to help services provide highly tailored support at the individual and group levels. Algorithms could even be trained to detect differences in practice and presenting crises across service modalities (eg, telephone, SMS text messaging, and web-based chat), flag features commonly present in repeat or unwelcome contacts to alert crisis supporters (particularly those still training or new) toward appropriate strategies and procedures to prevent burnout, and such AI-derived insights could be incorporated into staff training for quality assurance purposes. However, there are likely to be even greater concerns in the mental health field, as interpersonal communication and the therapeutic relationship between clients and service providers are critical.

This study aimed to address the significant gaps in understanding consumer perspectives of AI in mental health support for crisis support services by exploring, in the context of Lifeline, Australia’s largest crisis support helpline: (1) the level of support for the use of technology and automation, (2) the likelihood of service use if technology and automation were implemented, (3) the impact of demographic characteristics on the level of support and likelihood of service use if technology and automation were implemented, and (4) reasons for not using the services if technology and automation were implemented. These perspectives were explored for the Australian general community and specifically for Lifeline service users (help-seekers). It should be noted that AI can involve the automation of processes, such as self-driving vehicles, but automation does not necessarily include AI. The focus of this research is on AI-integrated technology and automation.

## Methods

### Design

A mixed methods approach using the triangulation design (validating quantitative data model [40]) was undertaken to explore consumer perspectives on the use of technology and automation in Lifeline’s crisis support services across 2 different samples of Australians (N=1853). First, a quantitative approach was used to establish the nature and range of participants’ levels of support for the collection of user information via AI and the likelihood of service use if technology and automation were implemented, followed by a qualitative exploration of the reasons provided by participants who were identified as not supporting or not likely to use Lifeline’s services. Owing to the paucity of precedent studies from which to determine the sample size for this research, the intended and achieved sample sizes were based on obtaining as large a sample as possible within the constraints of available project funding and timelines.

### Participants and Procedure

#### Sample 1—Community Sample

The community sample comprised a nationally representative sample of 1300 community-dwelling adults across Australia [38]. Respondents were aged 18 to 93 (mean 53.43, SD 18.49) years, and 52.8% (687/1300; valid percent) were women.

A computer-assisted telephone interview (CATI) was administered at the Social Research Centre at the Australian National University by trained interviewers. Data collection took place over 5 weeks, from October 28 to November 30, 2019. Contact details were purchased from the commercial sample provider SamplePages and included 16,245 mobile and 11,375 landline telephone numbers across Australia. The landline sample was stratified based on the state and capital city or rest of the state divisions. Geographic-based strata were not put in place for mobile devices, as no a priori geographic information was available. Random digit dialing (RDD) was used to obtain participants from all states or territories of Australia.

The interviews included 910 participants from the mobile RDD sample and 390 from the landline RDD sample. For people contacted on a landline number, any household member aged ≥18 years was eligible to participate. For people contacted on a mobile number, the survey was conducted with the phone user. Mobile phones were sent a pre-approach SMS text message with an opt-out option before contact by telephone. Interviews were conducted in English only. The average interview length was 14.8 minutes. There were no incentives for participation.

#### Sample 2—Help-Seekers

The help-seeker sample comprised 553 Lifeline help-seekers aged 18 to 77 (mean 39.60, SD 13.92) years, and 313 (74.2%; valid percent) were women.

A self-report survey was made available to Australian residents (aged ≥18 years) who had previously contacted Lifeline. Data collection took place over 6 months, from December 16, 2019, to June 16, 2020, via the web-based survey platform Qualtrics (Copyright 2021 Qualtrics) [41].

Recruitment was conducted through Lifeline Australia’s official social media pages (Facebook, Twitter, and LinkedIn) and website; a survey link shared at the end of Lifeline’s web-based chat and text message contacts; and snowballing across Lifeline Australia’s Lived Experience Advisory Group (LEAG) members and mental health organizations, such as Beyond Blue and SANE Australia. On clicking the survey link, participants were presented with an information sheet detailing the study aims, participant involvement, confidentiality and anonymity, data storage procedures, and investigator and ethics contact information. Informed consent was obtained from all participants through the web. Respondents were able to review and change their answers via a back button if desired.

The survey received 1278 total responses through Qualtrics, but 725 (56.73%) of them were <60% complete or the person had not previously contacted Lifeline. Analyses were compared with these cases excluded and included (using multiple imputation) when complete-case analysis was required. The median completion time was 11.7 minutes. No incentives were provided for participation.

### Measures

#### Overview

The questionnaire measures aimed to determine participants’ awareness, expectations, and outcomes of using Lifeline’s crisis support services. Demographic questions were asked about age, gender, sexual orientation, country of birth, main language spoken at home, indigenous status, and household composition. These characteristics were chosen because they represent groups of interest to Lifeline that may be at an elevated risk of suicidality and they can be used to assess regional variation. No standardized measures for assessing community or help-seeker expectations of AI as applied to crisis support services have been identified in the literature [42]; therefore, questions were developed by the research team in consultation with Lifeline and their LEAG. There were some minor differences in the questions between the CATI and web-based survey formats owing to the different nature of these data collection methods.

#### Support for Technology and Automation in Lifeline’s Crisis Support Services

Participants were asked, “When people contact Lifeline there is always a real person on the other end. However, there is the potential in the future for technology and automation to be used to help Lifeline counsellors to provide better services. Using a scale of 1 to 5, where 1 is not at all and 5 is very much, when people contact Lifeline, to what extent would you support Lifeline collecting information about individual users through technology and automation in order to tailor the services provided to the needs of each individual?” The additional prompts of “for example, to identify types of needs callers have and how they are feeling” and “automation refers to things like using artificial intelligence to monitor callers and measure their level of distress” were provided to sample 1 (community). In sample 2 (help-seekers) the following detail was provided: “for example, automation can refer to things like using artificial intelligence to monitor callers and measure their levels of distress.”

#### Likelihood of Service Use if Technology and Automation Were Used

Participants were asked, “If Lifeline were to use this type of technology and automation, do you think you would be less likely to use Lifeline, more likely to use Lifeline or would it not make a difference to you?” In sample 1 (community), the response options were 1 (less likely to use Lifeline), 2 (more likely to use Lifeline), and 3 (would not make a difference to you). In sample 2 (help-seekers), the response options were 1 (much less likely to use Lifeline), 2 (somewhat less likely to use Lifeline), 3 (neither more nor less likely to use Lifeline), 4 (somewhat more likely to use Lifeline), and 5 (much more likely to use Lifeline). For comparison between the samples, sample 2 scores were rescaled to range from 1 to 3, consistent with sample 1.

#### Reasons for Not Using the Lifeline Crisis Support Service if Technology and Automation Were Used

Participants from both samples who indicated that they would be less likely to use Lifeline were asked to elaborate on their response via the following open-ended question: “Why would you be less likely to use Lifeline as a result of Lifeline using this technology and automation?”

### Analysis

Quantitative data were analyzed using the statistical package SPSS (version 25.0; IBM Corporation) [43]. Descriptive statistics were computed for each measure and are reported as percentages. Demographics were compared across the 2 sample groups: sample 1 (community members) and sample 2 (help-seekers). To control for demographic differences between the samples, binary logistic regression models were used to determine the effect of sample type, while controlling for and assessing the impact of the demographic characteristics of age, gender, sexual orientation, country of birth, main language spoken at home, indigenous status, and whether living alone. Categorical data for age were further grouped into regression models to address the issue of small cell counts while broadly categorizing participants into young, middle-aged, and older adult groups for the interpretability of the results.

In the data set, 1.8%-9.7% data were missing at the variable level. Model estimates for each of the regression models were compared when missing data were excluded from the analysis using listwise deletion (the default treatment of missing data for SPSS logistic regression; N=1554-1573) and when missing data were included (N=1853) using SPSS’s multiple imputation of missing values to obtain pooled estimates across 40 imputations (m=40 number of imputations; refer to Multimedia Appendices 1 and 2 for multiple imputation results).

Significance was set at *P*<.01 to restrict significant effects to those that were more than trivial and provide an adjusted Cronbach *α* rate of *P*<.05 (based on the smallest sample for the help-seekers) [44,45]. Effect sizes were used as an additional criterion, with odds ratios of 1.52, 2.74, and 4.72 considered to be equivalent to Cohen *d* values of 0.2 (small), 0.5 (medium), and 0.8 (large), respectively [46].

Open-ended responses to the reasons question were analyzed in NVivo (version 12.0; QSR International [47]) by using thematic analysis, which is a method for identifying, analyzing, and reporting patterns in qualitative data. The coding and analysis of the responses for each sample were initially undertaken separately. In total, 2 coders undertook the coding, with cross-coding and discussion of themes until consensus was achieved. The themes from each sample were then considered together to identify common and unique themes across the samples. An essentialist or realist, inductive, and semantic approach was used to report the experiences, meanings, and reality of participants in ways that were explicitly linked to the data [48,49]. The 15-point Checklist of Criteria for Good Thematic Analysis by Braun and Clarke [50] was used in the transcription, coding, analysis, and written report processes by the authors (JSM and MO). The prevalence of themes was counted as the number of times a theme was evident across the data set. Selected data extracts representative of the main themes in each sample are presented in the Results section.

### Ethics Approval

This study was approved by the Human Research Ethics Committee of the University of Canberra (project ID: 2133).

## Results

### Overview

Descriptive information for the community and help-seeker samples is provided in Table 1. Comparatively, the community sample was significantly older and was more likely to have male and heterosexual participants. The help-seeker sample was younger and more likely to have participants who are female, speak only English at home, come from Australia, and live alone.

**Table 1:** Descriptive statistics for community and help-seeker samples.

		Community sample (n=1300)	Help-seeker sample (n=553)	χ^2^ or *t* (*df*)	*P* value	η^2a^ or Cramer *V*/Φ^b^
Age (years), mean (SD)		53.43 (18.49)	39.60 (13.92)	17.23 (1279.34)	*<.001* ^c^	*0.14*
**Gender, n (%)**				158.79 (2)	*<.001*	*0.30*
	Male	606 (46.72)	77 (18.2)			
	Female	687 (52.96)	313 (74.2)			
	Other	4 (0.30)	32 (7.6)			
**Sexual orientation, n (%)**				41.25 (1)	*<.001*	*0.15*
	Heterosexual	1167 (89.76)	302 (76.8)			
	Other	133 (10.23)	91 (23.2)			
**Country of birth, n (%)**				35.00 (2)	*<.001*	*0.14*
	Australia	961 (73.92)	346 (83.8)			
	Another English-speaking country	159 (12.23)	38 (9.2)			
	Non–English-speaking country	180 (13.84)	29 (7.0)			
**Main language** **spoken at home, n (%)**				7.49 (1)	*.006*	*0.68*
	English	1105 (85.06)	373 (90.5)			
	Other	194 (14.93)	39 (9.5)			
**Indigenous status, n (%)**				4.05 (1)	.04	0.05
	Yes	31 (2.40)	18 (4.5)			
	No	1259 (97.59)	382 (95.5)			
**Living situation, n (%)**				8.07 (1)	*.004*	−*0.07*
	Lives alone	248 (19.07)	107 (25.7)			
	Not alone	1052 (80.92)	309 (74.3)			

^a^η^2^=eta-squared measure of effect size.

^b^Φ=phi.

^c^*P* values <.01 are italicized.

### Support for Technology and Automation in Lifeline’s Crisis Support Services

**Figure 1:**
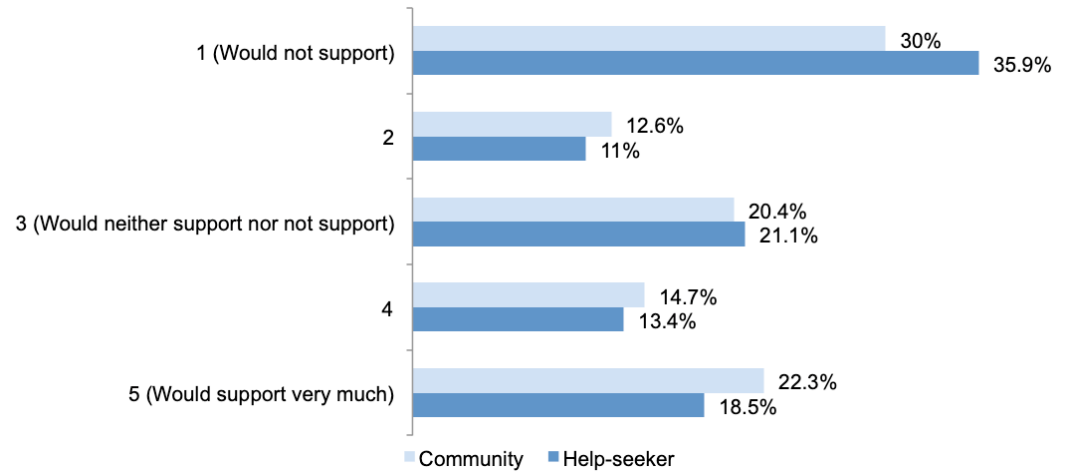
Levels of community (n=1268) and help-seeker (n=426) participants’ support in the use of technology and automation to tailor Lifeline’s crisis support service.

Figure 1 shows the percentage of participant support for the collection of user information to tailor Lifeline’s services. Overall, approximately one-third of the participants would not support the collection of user information, and approximately one-fifth of the participants would support it.

Given the demographic differences between the samples, a direct binary logistic regression was performed on participants’ level of support for the collection of user information to tailor Lifeline’s services, with sample type and 7 sociodemographic predictors included (age, gender, sexual orientation, country of birth, main language spoken at home, indigenous status, and whether living alone). A test of the full model with all 8 predictors against a constant-only model was statistically significant (N=1592, *χ^2^*_10_=23.4; *P*=.009). The model as a whole explained between 1.5% (Cox and Snell R-squared) and 2.0% (Nagelkerke R-squared) of the variance in support for collecting user information and correctly classified 57.66% (918/1592) of the cases. As shown in Table 2, only age significantly predicted participants’ level of support. Participants aged ≥35 years had at least 52% greater odds of reporting that they would not support the collection of user information (small effect) compared with those aged 18 to 34 years, controlling for all other factors in the model. Pooled estimates from the m=40 number of imputed data sets (N=1853) also found that age was the only significant predictor. Pooled odds for participants aged ≥35 years were slightly higher (54% vs 52%) but still represented a small effect (Multimedia Appendix 1).

**Table 2:** Logistic regression for support for the collection of user information to tailor Lifeline’s services (N=1592).

“Would not support”^a^		Odds ratio (99% CI)
Sample type (community)		1.16 (0.82-1.65)
**Age^b^ (years)**		
	≥55	1.55 (1.07-2.24)^c^
	35-54	1.52 (1.06-2.19)^d^
Gender (male)		1.11 (0.84-1.47)
Sexual orientation (heterosexual)		0.82 (0.54-1.26)
**Country of birth^e^**		
	Australia	1.13 (0.67-1.88)
	Another English-speaking country	1.34 (0.72-2.49)
Main language spoken at home (other than English)		1.12 (0.68-1.85)
Indigenous status (Aboriginal or Torres Strait Islander)		0.96 (0.42-2.19)
Living situation (lives alone)		1.22 (0.87-1.72)

^a^“Would support” combined with “Would neither support nor not support” is the reference group for comparison with “Would not support.”

^b^18 to 34 years is the reference group for age. Age groupings broadly reflect young adults (18-34 years), middle-aged adults (35-54 years), and older adults (≥55 years).

^c^*P*=.002.

^d^*P*=.003.

^e^Non–English-speaking country is the reference group for country of birth.

### Likelihood of Service Use if Technology and Automation Were Used

**Figure 2:**
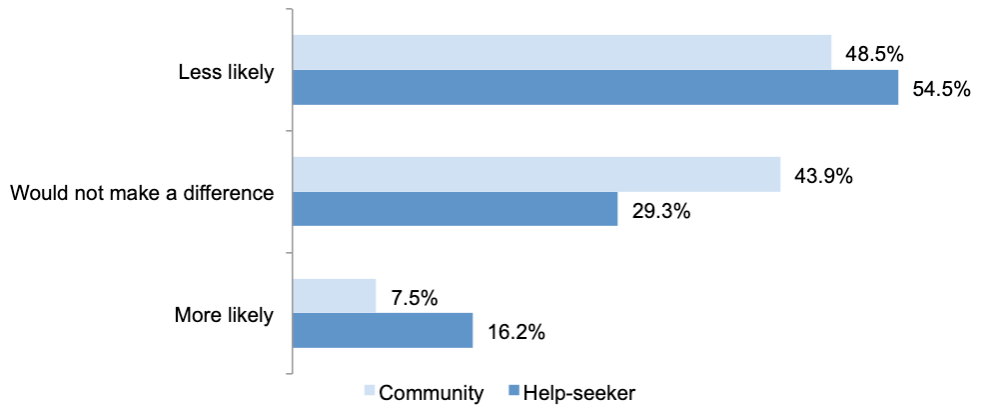
Likelihood of community (n=1247) and help-seeker (n=426) participants using Lifeline if technology and automation were used.

Figure 2 shows that approximately half of both samples stated that they would be less likely to use Lifeline if technology and automation were implemented, and only a minority would be more likely to use the service.

To test the sample effect while controlling for demographic differences, a direct binary logistic regression was performed. A test of the full model with all 8 predictors against a constant-only model was statistically significant (N=1572, *χ^2^*_10_=31.3; *P*=.001). The model as a whole explained between 2.0% (Cox and Snell R-squared) and 2.6% (Nagelkerke R-squared) of the variance in the likelihood of service use if technology and automation were implemented and correctly classified 54.33% (854/1572) of cases. As shown in Table 3, only age significantly predicted participants’ self-reported likelihood of service use. Participants aged ≥35 years had at least 48% greater odds of reporting that they would be less likely to use the service (small effect) than those aged 18 to 34 years, controlling for all other factors in the model. Pooled estimates from the m=40 imputed data sets (N=1853) also found that age was the only significant predictor. However, this effect was only observed at *P*<.01 for comparisons between participants aged ≥55 years and those aged 18 to 34 years (odds ratio 1.61, 99% CI 1.00-2.59; *P*=.009; Multimedia Appendix 2).

**Table 3:** Logistic regression for participants’ self-reported likelihood of service use if technology and automation were implemented at Lifeline (N=1572).

“Less likely”^a^			Odds ratio (99% CI)
Sample type (community)			1.23 (0.87-1.75)
**Age^b^ (years)**			
	≥55	1.66 (1.15-2.38)^c^	
	35-54	1.48 (1.03-2.12)^d^	
Gender (male)			1.27 (0.96-1.67)
Sexual orientation (heterosexual)			0.99 (0.65-1.50)
**Country of birth^e^**			
	Australia	1.36 (0.81-2.27)	
	Another English-speaking country	1.50 (0.80-2.80)	
Main language spoken at home (other than English)			0.78 (0.47-1.28)
Indigenous status (Aboriginal or Torres Strait Islander)			2.14 (0.89-5.16)
Living situation (lives alone)			1.25 (0.89-1.75)

^a^“More likely” combined with “Would not make a difference” is the reference group for comparison with “Would not support.”

^b^18 to 34 years is the reference group for age. Age groupings broadly reflect young adults (18-34 years), middle-aged adults (35-54 years), and older adults (≥55 years).

^c^*P*<.001.

^d^*P*=.005.

^e^Non–English-speaking country is the reference group for country of birth.

### Reasons for Not Using the Lifeline Crisis Support Service if Technology and Automation Were Used

**Figure 3:**
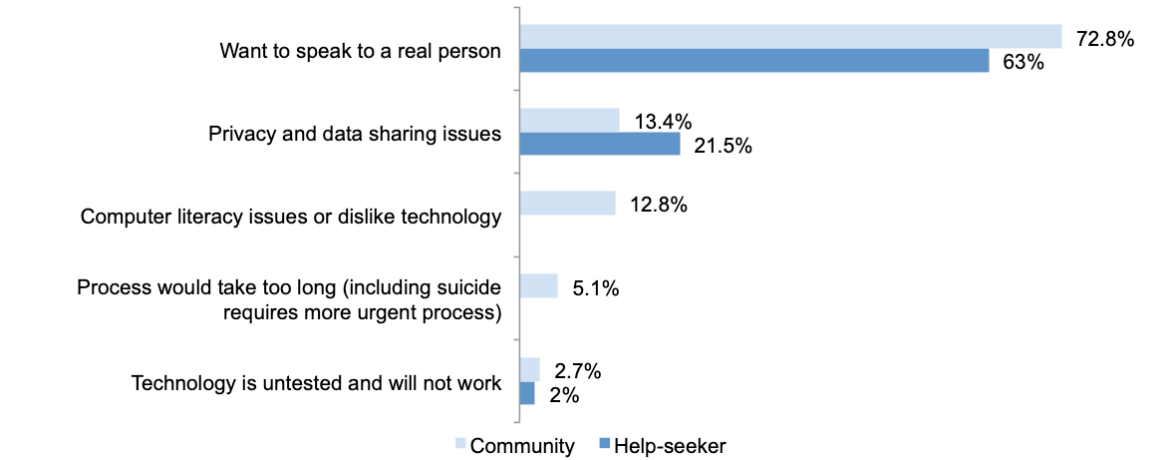
Reasons for community (n=595) and help-seeker (n=200) participants not using the Lifeline crisis support service if technology and automation were used—open-ended.

There were 837 community sample participants and help-seeker participants who indicated that they would be less likely to use Lifeline if technology and automation were used, and 94.9% (795/837) of the participants provided a qualitative response as to why (Figure 3). Participants could indicate more than one theme in their responses, resulting in a total response rate >100%. “General negative feedback about Lifeline,” “Positive feedback about Artificial Intelligence,” “Not sure,” and “Not applicable” responses make up the remaining percentage to 100% for the help-seeker sample. There were 3 common themes across the samples, and 2 were unique to the community sample.

### Common Themes

#### Want to Speak to a Real Person

Respondents overwhelmingly wanted to speak to a real person rather than a *robot* or *machine*. A particular concern was that human counselors would be replaced with an automated robot or machine services, which were expected to lack *heart*, *thought process*, *compassion*, and *understanding*. For example, a respondent stated the following:

You are talking to a robot, if I was suicidal, I would rather talk to somebody than [a] computer because the computer may not understand how you feel, but a person you’re talking to might have an idea of how to cope.

Community No 444, Male, 52 years of age

Another respondent said the following:

Because automation does not really understand people. Automation is just a script and people don’t like talking to machines.

Community No 235, Male, 45 years of age

Many emphasized the need for person-to-person contact and viewed this as a strength of the current Lifeline crisis support service:

Because I think one of the attractions of Lifeline is having a person at the other end, and I’d be concerned that AI [Artificial Intelligence] couldn’t pick up what I’m saying.

Community No 286, Female, 59 years of age

Another said the following:

I think Lifeline stands out because it’s always got a person there when so many other customer service interfaces are using technology—the reason I/they go to Lifeline is because of the person.

Community No 293, Male, 27 years of age

A total of 7 subthemes were identified as specific reasons for respondents wanting to speak to a real person. In the community sample, this included the lack of emotional connection (20/433, 4.6% of main theme), where respondents discussed how they would feel “less important” and “less connected” if technology and automation were used and how they would be left with “a perception that you might be wondering if you are more of a statistic than a person in need of help” (Community No 406, Male, 33 years of age). In the help-seeker sample, this included expectations that the experience would be impersonal (46/126, 36.5% of main theme), that human expertise is greater than what technology and automation could provide (30/126, 23.8%), that the use of technology and automation would be frustrating (9/126, 7.1%), that help-seekers require emotional connection (9/126, 7.1%), that help-seekers would feel devalued if technology and automation were used (6/126, 4.7%), and that only real people can provide comfort (4/126, 3.1%).

In relation to the expectation that the crisis support service would be impersonal, a respondent stated the following:

The distress and need is immediate. There is so much cold automation out there—sometimes the cause of our issues—the thought of more is depressing and sad. All we want is a human being. Some of us are minutes away from suicide. Don’t waste a second on bullshit automation. We need human beings.

Help-seeker No 205, Female, 49 years of age

Other respondents questioned how their interactions would differ from interacting with programmed devices:

Why would I want to talk to a computer instead of a person? I could use Siri or buy a Google home device and talk to it. What is the point of Lifeline if it becomes another computer to talk to?

Help-seeker No 38, gender not specified, 22 years of age

Many emphasized the limits of technology and that it could never replace human expertise. For example, a help-seeker stated the following:

Technology will never improve the human condition more than other humans can.

Help-seeker No 7, Female, 34 years of age

Another said the following:

AI [Artificial Intelligence] cannot sense a person’s level of distress and convey empathy the way a human can. When I hear someone say something that sounds automatic and stereotyped (reflections of strengths are a good example of this) I switch off and don’t feel able to engage with the person because I don’t feel they are listening. An AI service would do that to me—except all the time. There’s no one really listening and hearing me so there would be absolutely no point in calling. I’d feel worse after talking to an AI.

Help-seeker No 195, nonbinary, 48 years of age

Some respondents raised concerns about whether technologies such as AI could understand the nuances and complexities of help-seekers’ crises, particularly when this was already a difficult task for humans. For example, a respondent wrote the following:

I think there are things robots can do, but in my experience, understanding people is too complex even for humans.

Help-seeker No 401, gender not specified, 45 years of age

Help-seekers also noted the following perspectives:

AI would be based on a more generic format and would not consider the nuances of each particular concern and how the concerns affect people differently on any given day.

Help-seeker No 138, intersex, age not specified

Others indicated the following:

There is nothing more frustrating than being panicked or stressed and having to repeat yourself over and over again to a machine.

Help-seeker No 45, Female, 27 years of age

...when I’m depressed and/or suicidal, the last thing I need to deal with is automated phone “services,” when all I need to do is talk with a human.

Help-seeker No 65, Male, 54 years of age

Help-seekers emphasized that automation would only add to existing feelings of stress, particularly for older generations who may not be so familiar with the use of technology, and would result in many hanging up because “nobody wants to feel like they are a number instead of a person and that’s even more important when they are distressed” (Help-seeker No 443, Female, 49 years of age).

Finally, a few respondents specifically brought up the notion of comfort, with the perception that automation would take away the realness of contact. One help-seeker wrote the following:

Lifeline stands out as a service through which each caller speaks and connects directly to another person. There is intrinsic and immediate comfort in this, and the contact makes a huge difference to my confidence in the service.

Help-seeker No 1, Female, 55 years of age

#### Privacy and Data Sharing Issues

The next most common theme, although much less endorsed, was concerns with privacy and data sharing issues. Respondents were wary of scams, lack of security, and the potential of their data being used against them in the future. For example, a respondent said:

[I] do not like automation because so many scams rely on voice automation; it would make me stressed.

Community No 30, Female, 66 years of age

Another raised the issue of bias, limiting their trust in technology, stating the following:

Not something that I trust. Also don’t think it’s great considering these services are used by marginalised people. If the information, no matter how confidential, were leaked, it could be really bad for people who have already been dealt bad hands. The way those technologies are being developed and automated doesn’t seem to be going in a good direction. [I] believe that the development of these technologies can include biases, despite people believing that AI and tech is unbiased (built in bias).

Community No 588, Female, 26 years of age

Some were also concerned about feeling monitored, the level of control they would have over the information being shared and used, and the protection of their anonymity and confidentiality. A respondent said the following:

...some people experience paranoia in general and would be less likely to reach out if they felt they were being monitored in any way.

Help-seeker No 71, Female, 56 years of age

Another respondent stated the following:

Without specific details of the types of information to be collected and what would be done with that information, I am erring on the side of caution on this one. As a caller I like to be in control of the information I give out—I personally am quite an open-book anyway, so I generally don’t have a problem with sharing, but I think people need to feel trusted, and perhaps won’t feel trusted if this is implemented. I would also be concerned that the collection of this information would preference some callers over others somehow.

Help-seeker No 101, Female, 30 years of age

A respondent highlighted that this would be a particularly important consideration for vulnerable people:

...[they] are often abused. Using automation to detect distress could potentially cause an alert, which could put that person at risk of harm, abuse, and further trauma from services (eg, police, ambulance) when all they want is someone to listen. This type of “advancement” would be dangerous.

Help-seeker No 405, Female, 41 years of age

#### Technology Is Untested and Will Not Work

Concerns were also expressed regarding technology being something that was untested and may not work, which would exacerbate help-seekers’ levels of stress and anxiety. There were doubts about whether the machines could make judgments and accurately interpret mixed messages. A respondent said that it “can’t give instant answers” and involves “lots of hypotheticals” where “lots of things can change and a machine doesn’t know” (Community No 162, Male, 62 years of age). Another respondent stated the following:

I don’t believe automation can actually listen to a human being and understand the inflection and tones in the person’s voice. People who want to kill themselves don’t want to talk to automation. We hate when we speak to automation in other services (eg, banking); not good in this situation.

Community No 794, Female, 57 years of age

### Unique Community Themes

Community respondents provided 2 additional main themes (computer literacy issues or dislike of technology and the belief that the process would take too long) that were not evident in the help-seeking sample.

#### Computer Literacy Issues or Dislike of Technology

Community respondents spoke about how “frustrating” automation could be, particularly for older generations, as well as having a “hatred” or “aversion” to technology and robots. For example, a respondent stated the following:

Automation could be frustrating particularly if you’re not tech-savvy.

No 81, Female, 60 years of age

#### Process Would Take Too Long

A less common response was the belief that the automation process would take too long. Community respondents highlighted that “people using the service would want someone immediately on the line” and that help-seekers “could have hung up or would be feeling even more distressed by it than they already were” (No 116, Female, 31 years of age). A respondent indicated the following:

...it’s hard enough dealing with your emotions and figure out which number to press to get someone to be able to talk to you.

No 346, Female, 56 years of age

Reference was made to how this would be especially problematic for help-seekers who experience suicidality and require immediate support.

## Discussion

The aim of this mixed methods study was to understand the consumer perspectives of AI in mental health support from crisis support services. By surveying both general community members and Lifeline help-seekers, our results show a high level of resistance to and considerable misunderstanding of potential AI technologies in crisis line services.

### Principal Findings

Community and help-seeker participants were broadly consistent in their level of support and likelihood of service use if technology and automation were implemented in Lifeline’s crisis support services in Australia. One-third of the participants did not support the collection of information about individual users through technology and automation to tailor Lifeline’s services to individual needs, whereas approximately one-fifth of the participants were supportive. Approximately half of the participants reported that they would be less likely to use the Lifeline crisis support service if it implemented technology and automation. These findings reveal that the level of support for the use of technology and automation is not strong, that the likelihood of service use if technology and automation were implemented is not evident for most, that these views are evident across demographic groups, and that the reasons for not using the services if technology and automation were implemented are related to the preference for human contact and distrust of automation.

After controlling for demographic differences across the samples, older people (≥35 years) were found to have at least 48% greater odds of reporting that they would be less likely to support the collection of user information to tailor Lifeline’s crisis support services or to use these services if technology and automation were implemented compared with younger people. This finding may be attributed to young people, particularly men, who have higher levels of awareness, use, and acceptance of AI [32]. Younger people born after 1995 also belong to what is commonly referred to as the technological generation, with many *digital natives* spending at least nine hours a day interacting in digital environments [51]. As such, the promotion of AI acceptance in crisis support service contexts may be needed more in middle- and older-aged people, many of whom would not have grown up with the same experiences of technology as their younger counterparts. The multiple imputed data analysis corroborated these findings, with the exception of the age effect for being significantly less likely to use the service only applying to people aged ≥55. However, it is important to highlight that imputed values may not accurately represent the actual percentage of self-reported likelihood of service use because these values are not obtained from *real* consumers.

Importantly, we found that community and help-seeker participants strongly held assumptions that the use of *technology* and *automation* in crisis support would involve the replacement of human counselors with automated robots or machine services, although the questionnaire clearly stated “however, when people contact Lifeline there is always a real person on the other end.” This finding shows that the replacement of people-centered services with robots and machines is a real fear for consumers. This may be attributed to people obtaining much of their understanding from popular media (ie, films [52]) or past negative experiences with common automated services such as banking (which was a comparison noted by many participants) or the very poorly received Australian debt recovery program, Robodebt [53]. Such preconceptions about automation clearly had a major impact on the reasons community and help-seeker participants provided for not using Lifeline’s services if technology enhancements were introduced, which would need to be carefully addressed if AI is to be used effectively to support human decision-making processes in crisis support contexts.

Specifically, “want to speak to a real person” and “privacy and data sharing issues” were the most commonly reported main themes and concerns among both community members and help-seekers. For help-seekers, wanting to speak to a real person was attributed to participants believing that the human element is essential because human expertise is greater than what technology and automation could provide, that the use of technology and automation would be frustrating, that help-seekers require emotional connection and would feel devalued if technology and automation were used, and that only real people can provide comfort. Regarding confidentiality issues, community members were wary of scams, lack of security, and the potential of their data being used against them in the future, which are concerns related to the risks of technology use in general. Help-seekers were more concerned about feeling monitored, the level of control they would have over the information being shared and used, and the protection of their anonymity and confidentiality, particularly for vulnerable people such as those who have experienced abuse.

### Study Implications

These findings show the need for clear communication and education about the potential use and benefits of AI in crisis support services, particularly to assuage fears regarding the replacement of counselors and removal of human-centered care, as well as transparency around confidentiality and how individuals’ data are collected, used, and stored so that trust is not eroded [54]. It has been highlighted that even for research in this area, more explicit consideration of the ethical and legal issues in current and future research on algorithmic and data-driven technologies in mental health initiatives is required [55].

Overall, community and help-seeker participants’ levels of support for technology and automation largely align with previous research conducted in medical health contexts. The results are consistent with the *uniqueness neglect* psychological driver, as participants strongly felt that only another human could understand the circumstances and nuances of another human, supporting this issue as an important target for consumer education about the role of AI [29]. In particular, strong negative attitudes from prior experiences of automation that were frustratingly unresponsive to human needs (such as banking and government services) will need to be redressed. Attention to involving consumers in AI research and educating them about potential implementation are critical priorities. Such efforts could be used to help train and prepare crisis support professionals for the inevitable use of new technologies, such as AI, in their services, but also extend to potential consumers, funders, and decision makers to ensure that all stakeholders understand how AI can be used to enhance existing services to continue to support, not replace, human connections and decision-making in ethical ways.

Notably, despite the resistance of about half of the participants to using the service if automation was implemented, the other half said that their decision would be unaffected. Of these, approximately one-tenth reported that they would be more likely to use the service, highlighting the scope for endeavors that aim to promote the acceptability of AI in crisis support services. However, given the paucity of existing research in this area, more quantitative and qualitative studies are needed to better understand why consumers would and would not support the use of AI in their mental health and crisis support services. Research needs to identify the barriers and facilitators to the acceptance of AI and inform the development of AI awareness and promotion education initiatives to modify fear-based or inaccurate assumptions about the role, application, and impact of AI on personal user experiences in mental health support. Our research shows that preconceived notions, such as fears of talking to a *robot*, are pervasive and that the ways in which AI can be implemented to substantially improve the help-seeking experience are not well understood.

### Strengths and Limitations

The strengths of this study include the large nationally representative community sample and large help-seeker sample used to address the study aims and the use of multivariate analyses, which enabled the examination of the extent to which demographic factors impacted consumer perspectives of AI in relation to Lifeline’s crisis support service. This study had several limitations. First, with the lack of standardized measures for assessing community help-seeker expectations of AI as applied to crisis support services, support for technology and automation and likelihood of service use were assessed using 2 single-item measures developed in consultation with Lifeline and their LEAG. Although research has shown that single-item measures can perform well relative to their full scales across psychological, health, and marketing research [56,57], it is noted that reliability and validity information for the developed measures is not currently available. Psychometric research is needed to further develop and refine effective measures for assessing consumer expectations in this space.

Second, the depth of the qualitative thematic analysis was restricted to the format of the questions and the inductive approach used, limiting interpretative power beyond the surface descriptions provided by community members and help-seekers. Respondents may have endorsed additional themes if they had been probed specifically about their views and had the opportunity to elaborate. The lack of in-person and group discussions may have also reduced the richness of the qualitative data obtained, although this was mitigated by obtaining data from such large samples. Future research should incorporate in-depth focus groups to explore consumers’ reluctance to approve technologically enhanced crisis support services.

Third, the study only focused on why participants would be less likely to use Lifeline’s services if technology and automation were used and not on why they would be more likely to do so, which could include faster response times, higher quality interactions, fewer missed calls, and greater capacity to support the community. The explicit form and role of technology and automation in Lifeline’s services were also not fully preempted by participants when asking about their reasons for not using Lifeline’s services, which may have led to many assuming technology and automation to be relatively extreme and intrusive. We found it difficult to simply and clearly contextualize the relevant questions in a survey format. Explaining the potential uses of AI and debunking myths about automation are difficult without unduly influencing participants’ responses, particularly given the complex nature of AI and ML innovations. Nevertheless, future studies would benefit from providing additional framing and specificity around concepts of technology and automation (ie, that human counselors are not being replaced by robots or machines) and incorporating positive reasons for use, which would enable investigation into both the barriers and facilitators of AI-integrated service use in mental health and crisis support contexts.

Fourth, there were significant demographic differences between the 2 samples and different data collection methods were used. For example, men were underrepresented in the help-seeker sample. Although the sample differences were statistically controlled for, other confounding factors may have impacted the results. Finally, this research cannot ascertain causality regarding the link between beliefs and actual help-seeking behavior, and, as such, the integration of technology and automation in services may not result in actual crisis support service use refusal.

### Conclusions

To our knowledge, this is the first mixed methods study to explore consumer perspectives of AI in mental health, specifically regarding its application in crisis support services. As such, this study addresses a significant knowledge and practice gap in relation to consumers’ acceptance of new technologies in response to the rapid advancement of technology use in health and mental health care and support. Although some level of consumer support exists for the collection of user information to tailor services via technology, the majority were reluctant to use AI-integrated crisis support services. Greater reluctance was evident among older people. Addressing community and help-seeker concerns about AI in mental health support contexts, including emphasizing how technology will augment rather than replace human connection and decision-making, with the goal of positively and ethically supporting service users’ experiences, is of high priority given that these groups are the ultimate consumers of AI. Those most affected, namely, service users and their service providers, need to be fully involved in the development and implementation of innovative technologies to ensure they are appropriately designed and effectively adopted to improve mental health and crisis support services in the near future and beyond. However, the value of the human connection factor should not be lost.

## Appendix

Multimedia Appendix 1 Logistic regression on multiple imputed data (m=40) for support for the collection of user information to tailor Lifeline’s services (N=1853).

Multimedia Appendix 2 Logistic regression on multiple imputed data (m=40) for participants’ self-reported likelihood of service use if technology and automation were implemented at Lifeline (N=1853).

## Abbreviations

AI: artificial intelligence
CATI: computer-assisted telephone interview
LEAG: Lived Experience Advisory Group
ML: machine learning
RDD: random digit dialing
