# Supplementary material for: Consumer Perspectives on the Use of Artificial Intelligence Technology and Automation in Crisis Support Services: Mixed Methods Study
Source: JMIR Hum Factors. 2022 Aug 5;9(3):e34514. doi: 10.2196/34514 (PMC9391967; doi:10.2196/34514)
Supplement: Multimedia Appendix 1 [file humanfactors_v9i3e34514_app1.docx]

## Multimedia Appendix 1

**Table S4.** Logistic regression on multiple imputed data (m=40 datasets) for support for the collection of user information to tailor Lifeline’s services (N=1853).

| “Would not support”^a^ | | Odds ratio (99% CI) |
| --- | --- | --- |
| Sample type (community) | | 1.31 (0.76-2.27) |
| **Age^b^ (years)** | |  |
|  | ≥55 | 1.65 (1.07-2.54)^c^ |
|  | 35-54 | 1.54 (1.02-2.32)^d^ |
| Gender (male) | | 1.12 (0.79-1.59) |
| Sexual orientation (heterosexual) | | 0.93 (0.46-1.88) |
| **Country of birth^e^** | |  |
|  | Australia | 1.06 (0.44-2.57) |
|  | Another English-speaking country | 1.34 (0.39-4.52) |
| Main language spoken at home (other than English) | | 1.14 (0.54-2.40) |
| Indigenous status (Aboriginal or Torres Strait Islander) | | 0.75 (0.11-5.04) |
| Living situation (lives alone) | | 1.21 (0.73-1.99) |

^a^“Would support” combined with “Would neither support nor not support” is the reference group for comparison with “Would not support.”

^b^18 to 34 years is the reference group for age. Age groupings broadly reflect young adults (18-34 years), middle-aged adults (35-54 years), and older adults (≥55 years).

^c^*P*=.003.

^d^*P*=.006.

^e^Non–English-speaking country is the reference group for country of birth.
