# Supplementary material for: Consumer Perspectives on the Use of Artificial Intelligence Technology and Automation in Crisis Support Services: Mixed Methods Study
Source: JMIR Hum Factors. 2022 Aug 5;9(3):e34514. doi: 10.2196/34514 (PMC9391967; doi:10.2196/34514)
Supplement: Multimedia Appendix 2 [file humanfactors_v9i3e34514_app2.docx]

## Multimedia Appendix 2

Table S5. Logistic regression on multiple imputed data (m=40 datasets) for participants’ self-reported likelihood of service use if technology and automation were implemented at Lifeline (N=1853).

| “Less likely”^a^ | | Odds ratio (99% *CI*) |
| --- | --- | --- |
| Sample type (community) | | 1.41 (0.91-2.20) |
| **Age^b^ (years)** | |  |
|  | ≥55 | 1.61 (1.00-2.59)^c^ |
|  | 35-54 | 1.46 (0.94-2.25)^d^ |
| Gender (male) | | 1.22 (0.84-1.79) |
| Sexual orientation (heterosexual) | | 1.00 (0.52-1.93) |
| **Country of birth^e^** | |  |
|  | Australia | 1.23 (0.44-3.42) |
|  | Another English-speaking country | 1.42 (0.40-4.95) |
| Main language spoken at home (other than English) | | 0.92 (0.42-2.00) |
| Indigenous status (Aboriginal or Torres Strait Islander) | | 1.34 (0.22-8.11) |
| Living situation (lives alone) | | 1.20 (0.75-1.92) |

^a^“More likely” combined with “Would not make a difference” is the reference group for comparison with “Would not support.”

^b^18 to 34 years is the reference group for age. Age groupings broadly reflect young adults (18-34 years), middle-aged adults (35-54 years), and older adults (≥55 years).

^c^*P*<.009.

^d^*P*=.023.

^e^Non–English-speaking country is the reference group for country of birth.
